# Task shifting: the answer to the human resources crisis in Africa?

**DOI:** 10.1186/1478-4491-7-49

**Published:** 2009-06-21

**Authors:** Uta Lehmann, Wim Van Damme, Francoise Barten, David Sanders

**Affiliations:** 1 School of Public Health, University of the Western Cape, Bellville, South Africa; 2 Department of Public Health, Institute of Tropical Medicine, Antwerp, Belgium; 3 International Health, Department of Primary Care and Community Care, Radboud University Nijmegen, the Netherlands

## Abstract

Ever since the 2006 *World Health Report *advocated increased community participation and the systematic delegation of tasks to less-specialized cadres, there has been a great deal of debate about the expediency, efficacy and modalities of task shifting.

The delegation of tasks from one cadre to another, previously often called substitution, is not a new concept. It has been used in many countries and for many decades, either as a response to emergency needs or as a method to provide adequate care at primary and secondary levels, especially in understaffed rural facilities, to enhance quality and reduce costs. However, rapidly increasing care needs generated by the HIV/AIDS epidemic and accelerating human resource crises in many African countries have given the concept and practice of task shifting new prominence and urgency. Furthermore, the question arises as to whether task shifting and increased community participation can be more than a short-term solution to address the HIV/AIDS crisis and can contribute to a revival of the primary health care approach as an answer to health systems crises.

In this commentary we argue that, while task shifting holds great promise, any long-term success of task shifting hinges on serious political and financial commitments. We reason that it requires a comprehensive and integrated reconfiguration of health teams, changed scopes of practice and regulatory frameworks and enhanced training infrastructure, as well as availability of reliable medium- to long-term funding, with time frames of 20 to 30 years instead of three to five years. The concept and practice of community participation needs to be revisited.

Most importantly, task shifting strategies require leadership from national governments to ensure an enabling regulatory framework; drive the implementation of relevant policies; guide and support training institutions and ensure adequate resources; and harness the support of the multiple stakeholders. With such leadership and a willingness to learn from those with relevant experience (for example, Brazil, Ethiopia, Malawi, Mozambique and Zambia), task shifting can indeed make a vital contribution to building sustainable, cost-effective and equitable health care systems. Without it, task shifting runs the risk of being yet another unsuccessful health sector reform initiative.

## Introduction

Ever since the 2006 *World Health Report *advocated the systematic delegation of tasks to less-specialized cadres and "placing strong emphasis on patient self-management and community involvement" [1], there has been a great deal of debate about the expediency, efficacy and modalities of task shifting.

The delegation of tasks from one cadre to another, previously often called substitution [2], is not a new concept. It has been used in many countries and for many decades, either as a response to emergency needs or as a method to provide adequate care at primary and secondary levels, especially in understaffed rural and urban facilities, to enhance quality and reduce costs [3]. However, rapidly increasing care needs generated by the HIV/AIDS epidemic and accelerating human resource crises in many African countries, often within a context of near-collapse of public health systems and increasing health inequalities within and between countries, have given the concept and practice of task shifting new prominence and urgency. For example, it is estimated that sub-Saharan African countries will have to triple their current health workforce in order to come close to reaching the Health Millennium Development Goals [4].

But while the above factors have lent urgency to task shifting debates, particularly in many southern African countries, the question arises as to whether task shifting and increased community participation can be more than a short-term solution to address the HIV/AIDS crisis and can contribute to a revival of the primary health care approach as an answer to health systems crises [5].

## Discussion

Reviews of evidence consistently show that delegation of tasks, whether from doctors to non-physician clinicians, including nurses [2,6-14], from nurses to nursing assistants or aides or to non-professional or lay health workers and patients [14-17] can lead to improvements in access, coverage and quality of health services at comparable or lower cost than traditional delivery models [2,18].

The literature is also unanimous, however, that any long-term success of task shifting hinges on serious political and financial commitments. Task shifting requires careful attention to organization, structure and resourcing of health services. Samb et al. argued, in the context of HIV/AIDS services, that task shifting "must be aligned with the broader strengthening of the health system if it is to prove sustainable". They called on governments and international and bilateral agencies to help prepare health systems to successfully implement task shifting by ensuring the establishment of appropriate regulatory frameworks and the building of training and management capacity [3]. Given the comparably poor record of initiatives to strengthen health systems and to enhance capacity in many African countries, the question has to be asked: What does this mean?

Crucially, it requires the integration of the concept and roles of new cadres, changed scopes of practice and regulatory frameworks, enhanced training infrastructure, etc., into the mainstream health system, and a systematic engagement with all the consequences. The delegation of voluntary counselling and testing to lay health workers, for example, may require not only the recruitment and training of lay health workers, but also changes to the roles, skills and workloads of nurses who have to coordinate and supervise them [19].

Ultimately, successful task shifting requires a comprehensive and integrated reconfiguration of health teams, particularly at community and primary care levels. Without a health team approach, the introduction of new cadres or delegation of tasks will invariably remain a fragmented and unsustainable "add-on".

An example of a large-scale reconfiguration of health teams is the "family health teams" created in Brazil in the early 1990s. These teams, under the *Programa de Sáude da Família*, usually are composed of at least one physician, one nurse, a nurse assistant and (usually) four or more community health workers who take responsibility for providing a broad range of primary health care services in an assigned geographical area [20]. It is worthwhile noting that this programme is a central component of a broader and well-funded new health policy and is embedded within a comprehensive approach at area level that also involves community-based organizations in decision-making about allocation of resources and in actions that address some upstream determinants of health at the local level.

Furthermore, the introduction and/or integration of new cadres and community members into health care delivery requires the availability of reliable medium- to long-term funding, with time frames of 20 to 30 years instead of three to five years [21]. Not only remuneration, but funding for training, supervision and infrastructure support must be ensured. This becomes particularly challenging when the targets for these initiatives are often remote, hard-to-reach and notoriously underresourced areas in countries with fiscal crises.

The concept and practice of community participation as an important ingredient of task shifting, particularly with regard to community health workers, needs to be revisited. While the importance and benefit of community involvement has long been acknowledged, it is nevertheless widely recognized that a considerable gulf exists between the ideal of programmes driven and owned by communities and programme realities. It is further agreed that while there are few success stories of lasting community participation, sustainability and impact of programmes require the ownership and active participation of communities as a non-negotiable precondition [22-27].

If community involvement is to move from rhetoric to reality and from specific, often fragmented, initiatives to everyday practice, certain key issues need to be addressed. Findings from research highlight "the importance of commitment, the need to be clear about the levels and extent of participation, and the importance of resolving matters of representation" [28].

Involving lay health workers or community health workers in health care delivery can enhance community participation considerably, if these nonprofessional workers are genuine representatives of their communities and give these communities voice within health systems. Many authors have indicated that these dimensions are central to any discussion of participatory processes whatever the context – although some contexts are more favourable than others for genuine participation [29] – and that some degree of clarity is called for when facilitating community involvement [22,30-32].

Lastly, and probably most importantly, any serious commitment to task shifting requires leadership from national governments. It is national government's role to ensure an enabling regulatory framework and credentialing system, to drive the implementation of relevant policies and to resource, guide and support training institutions to not only upgrade training but also ensure appropriate initial and continuing education (integrated, multidisciplinary, community- and outcomes-based).

Crucially, the national government must harness the support of the multiple stakeholders who affect and are affected by the reconfiguration of tasks (such as professional bodies and associations; trade unions; ministries of health, education, finance and public service; nongovernmental and community organizations; and local health structures) [33]. Where this is not the case, task shifting will exist on the political and organizational periphery of the formal health system, exposed to policy and funding fashions, and become fragile and unsustainable.

In this context, it is noteworthy that in several countries, task shifting has been enthusiastically taken up by NGOs, with strong community links at the local level, but with limited potential for national scaling-up, as their presence is often geographically circumscribed and may often be temporary, depending often on external, short-term donor funding.

Task shifting, while driven by the urgencies of conquering the HIV/AIDS epidemic, holds the potential of enabling countries to build sustainable, cost-effective and equitable health care systems, thus moving closer not only to the Millennium Development Goals, but also the elusive Health for All goal. However, the challenge of achieving success cannot be underestimated. It requires a willingness to learn from those with relevant experience (such as Brazil, Ethiopia, Malawi, Mozambique and Zambia) and to suspend conventional (and often conservative) wisdom on what can and cannot be done in favour of creativity and problem-solving.

## Competing interests

The authors declare that they have no competing interests.

## Authors' contributions

All authors jointly conceived of the article. UL had primary responsibility for the initial draft of the manuscript. UL, WVD, FB and DS all contributed substantially to the intellectual content, writing and finalization of the manuscript. All authors read and approved the final manuscript.
